# Treatment of Intermittent Fevers by a Single Dose of Quinine

**Published:** 1850-09

**Authors:** 


### ARTICLE II.

### *Treatment of Intermittent Fevers by a single dose of Quinine.*

Dr. Pfeufer, of Heidelberg, states he has had much opportunity of treating this disease, and was formerly in the habit of prescribing from 15 to 20 grains, in divided doses, in the intervals of the paroxysms. Latterly he had given five-grain doses, until from 40 to 60 grains were taken, and with great success. The number of patients having greatly increased during the bivouacs consequent upon the revolutionary disturbances, the expense of so much Quinine was found a serious consideration, and he determined to try whether by a different mode of administration less might not suffice, and certainly, if the results he has arrived at are confirmed by others, he will have conferred no ordinary boon on the distributors of charitable medical relief. He finds, indeed, not only that the expense may be vastly diminished, but the cure expedited and rendered more certain, by administering a *single ten-grain dose* (made into four pills, with ext. of *millefolium*), on a day free from Fever. This dose is well borne, none of the inconveniences which result from the long-continued use of small doses, or the tinitus, giddiness, &c., produced by very large ones, presenting themselves. The subsequent attack is weaker, and its successors still more so, the convalescent remaining in the hospital from four to eight days. A tabular view of the particulars of 34 cases so treated is given.---*Henle and Pfeufer's Zeit.*---*Brit. and For. Medico-Chirurg. Review*, April, 1850, p. 534.

